# The relationship between occupational noise and vibration exposure and headache/eyestrain, based on the fourth Korean Working Condition Survey (KWCS)

**DOI:** 10.1371/journal.pone.0177846

**Published:** 2017-05-23

**Authors:** Jihyun Kim, Wanhyung Lee, Jong-Uk Won, Jin-Ha Yoon, Hongdeok Seok, Yeong-Kwang Kim, Seunghyun Lee, Jaehoon Roh

**Affiliations:** 1 The Institute for Occupational Health, University College of Medicine, Yonsei University, Seoul, Republic of Korea; 2 Graduate School of Public Health, College of Medicine, Yonsei University, Seoul, Republic of Korea; 3 Incheon Worker’s Health Center, Incheon, Republic of Korea; 4 Department of Preventive Medicine, College of Medicine, Yonsei University, Seoul, Republic of Korea; 5 The First Department of Internal Medicine, School of Medicine, University of Occupational and Environmental Health, Kitakyushu, Japan; Taipei Veterans General Hospital, TAIWAN

## Abstract

**Introduction:**

The individual and combined effect of occupational noise and vibration exposures, on workers’ health has not been thoroughly investigated. In order to find better ways to prevent and manage workers’ headache, this study aimed to investigate the effects of occupational noise and vibration exposure on headache/eyestrain.

**Methods:**

We used data from the fourth Korean Working Condition Survey (2014). After applying inclusion and exclusion criteria, 25,751 workers were included. Occupational noise and vibration exposure and the prevalence of headache/eyestrain were investigated by self-reported survey. Chi-square tests were used to compare differences in baseline characteristics between the group with headache/eyestrain and the group without. Odds ratios and 95% confidence intervals were estimated using a logistic regression model adjusted for several covariates. Area under the receiver operating characteristics curve (AUROC) analysis was used to evaluate the effect of occupational noise and/or vibration exposure.

**Results:**

Among the 25,751 study subjects, 4,903 had experienced headache/eyestrain in the preceding year. There were significant differences in age, education level, household income, occupational classification, shift work, occupational vibration exposure, and occupational noise exposure between the two groups (all p<0.05). The odds ratios between each exposure and headache/eyestrain increased proportionally with the level of exposure, increasing from 1.08 to 1.26 with increasing vibration exposure, and from 1.25 to 1.41 with increasing noise exposure. According to the AUROC analysis, the predictive power of each exposure was significant, and increased when the two exposures were considered in combination.

**Discussion:**

The findings of this study show that both occupational noise and vibration exposures are associated with headache/eyestrain; noise exposure more strongly so. However, when the two exposures are considered in combination, the explanatory power for headache/eyestrain is increased. Therefore, efforts aimed at reducing and managing occupational noise and vibration exposure are crucial to maintaining workers’ health.

## Introduction

Modern society has undergone remarkable economic and social growth over recent years, with a corresponding desire for better living environments. Concurrently, disease prevention efforts and improvements to the total workplace environment have increased. Furthermore, the concept of well-being has widened to include the systemic effects of occupational toxicology; not only the traditional target organ effects. For example, noise-induced hearing loss was considered the most important issue in the workplace; recently, however, noise-induced systemic effects, such as cardiovascular, neurological, and psychological diseases have been also investigated.

Occupational noise and vibration exposures have not been thoroughly investigated because noise and vibration come from various sources [1–4] and researchers face difficulties in systematically identifying and managing risks associated with these two factors. Furthermore, subjective responses to noise and vibration cannot be quantified easily, adding to the difficulties experienced by researchers in measuring their levels and investigating their effects. However, considering the associations between various disorders and noise and vibration [5,6], these two occupational exposures should be at the center of academic interest. Therefore, studies on the effects of noise and vibration exposure are vital.

It is difficult to consider headache and eyestrain separately since the two symptoms are closely related. Previous studies in the field of ophthalmology concluded that eyestrain is usually accompanied by headache[7] through various channels. Vincent et al. suggested that factors such as blurred vision and dryness related to eyestrain tend to precipitate headache[8]. Carruthers et al. postulated a mechanism for the relationship between eyestrain and headache in terms of abnormal contraction of the orbital portion of the orbicularis oculi muscle[9], while functional abnormalities of accommodative and convergence mechanisms have also been considered as a closely linking mechanism to headache and eyestrain[10].

Although recent studies are beginning to report an association between headache/eyestrain and noise or vibration exposure, the importance of this has not been fully studied. Almost all previous studies have considered occupational noise exposure and occupational vibration exposure separately. Also, given the burden of headache/eyestrain [11], various research approaches are necessary.

In order to find better ways to prevent and manage workers’ headache/eyestrain, we aimed to investigate the association between noise and vibration exposure, and headache. Unlike previous studies that focused merely on the correlation between headache and each of the two risk factors individually, this study aimed to provide information about the combined effect of occupational noise and vibration exposures and to determine which of the two risk factors has a greater impact on headache/eyestrain.

## Methods

### Study design and participants

We used data from the fourth Korean Working Condition Survey (KWCS) in 2014 conducted by the Korea Occupational Safety and Health Agency. A total of 50,007 working individuals were surveyed and interviewed by multi-area random sampling to ensure that the sample was representative of active Korean workers aged >15 years. All respondents also agreed to participate in further scientific research and were assigned a randomly selected participant number to protect their anonymity. The quality of the Korean Working Condition Survey was assured and the survey was deemed valid and reliable; the well-organized random sampling procedure and the well-designed questionnaire used contributed to quality assurance [12]. Furthermore, in another study, the reported work-related symptoms in KWCS were evaluated by comparison with those used in the European Working Conditions Survey (EWCS). According to that study, the most common symptoms in Korea were muscular pain (18.1%), followed by stress, backache, fatigue, and headache (11.2%). Moreover, the rank of symptoms in KWCS was very similar to those in EWCS [13]. The data used for our study were presented in S1 File.

**Fig 1:**
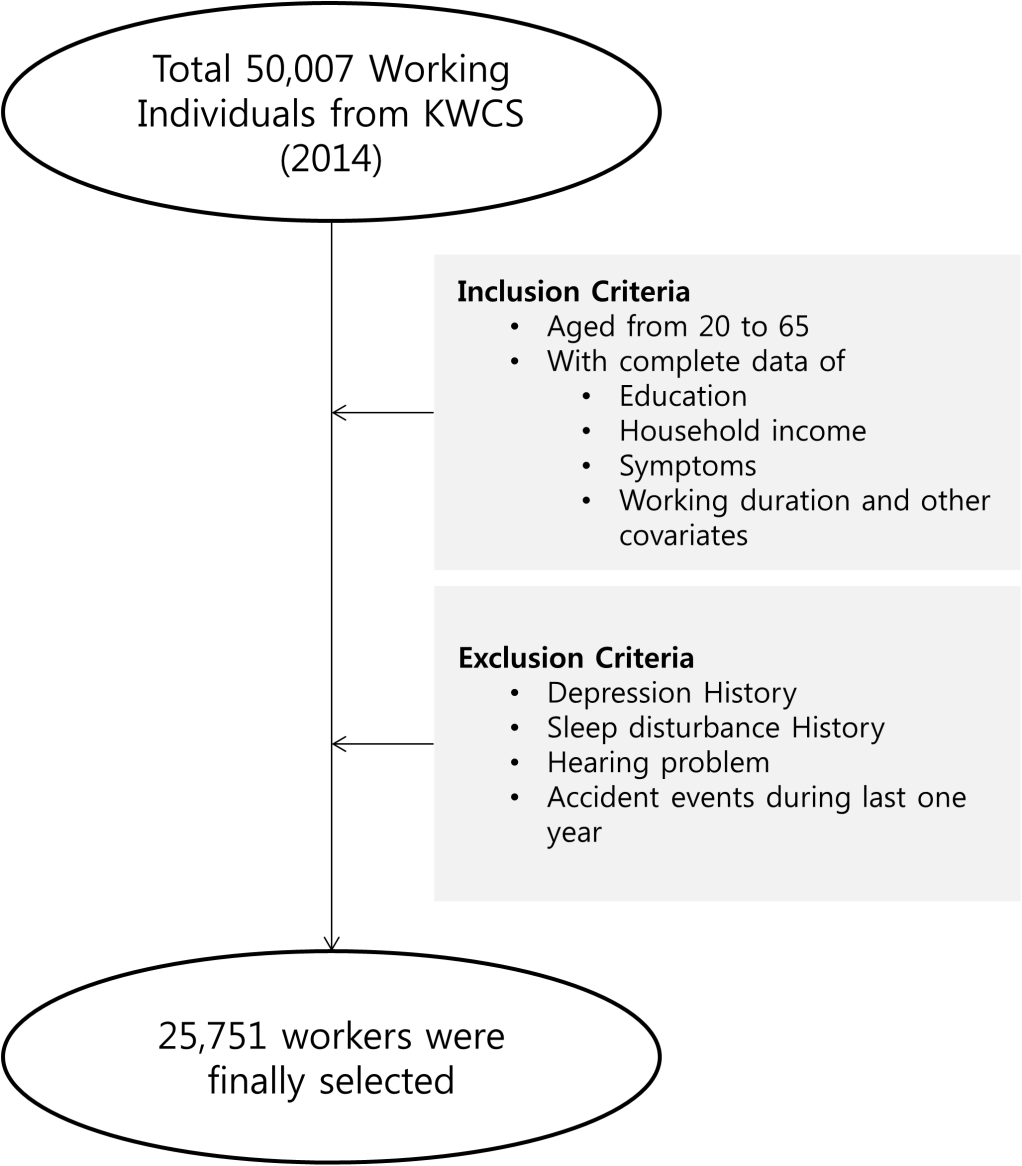
Process of participant enrolment.

First, we selected 27,485 subjects aged 20–65 years with complete data regarding education, household income, symptoms, working duration, and other covariates (Fig 1). We excluded individuals who suffered from depression or sleep disturbance, or who had been involved in accidents during the preceding year as these factors are associated with both headache and eyestrain [14–16]. We also excluded those with hearing disorders as such disorders may act as obstacles to assessing occupational noise exposure. Thus, a total of 1,734 subjects were excluded from our study, leaving 25,751 workers (22,245 men and 3,506 women) included for analysis in the present investigation.

### Main variables

Health problems were diagnosed using a self-reported questionnaire that enquired about symptoms. The presence of headache or eyestrain was assessed via response to the question: “Over the last 12 months, did you have any of the following health problems?” This was identical to the question used in the EWCS. Additionally, subjects were asked the following questions regarding occupational exposure: “In your workplace, are you exposed to noise so loud that you have to raise your voice to keep a conversation during work?” and “In your workplace, are you exposed to vibrations from hand tools and machinery?” A previous study estimated the accuracy of the responses regarding perceived noise to have a sensitivity of 68.4% and specificity of 74.6% at a noise exposure of 85 dB (as measured by a noise dosimeter) [17]. Another study investigated vibration perception thresholds in exposed workers, and found the optimal limit to be 138dB (sensitivity 63%; specificity 86%) at 500 Hz [18]. Our participants could subjectively answer each question according to a seven-point scale (all of the time, almost all of the time, approximately 3/4 of the time, approximately 1/2 of the time, approximately 1/4 of the time, almost never, never). These responses were divided into three categories: none (“never”), mild (“almost never” or “approximately 1/4 of the time”), and severe (“approximately 1/2 of the time” or more frequently). Participants were classified into two groups, cases and controls, according to whether they had experienced headache or eyestrain during the preceding 12 months.

### Covariates

Potential confounding variables included sex, age, educational level, and household income. Occupational characteristics included occupational classification, shift work (yes or no); working hours (< 48 vs. ≥ 48 hours per week); and the use of personal protective equipment (PPE), such as ear plugs, helmets, or safety goggles. Occupational classifications were regrouped into three of the ten major categories of the International Standard Classifications of Occupations, according to skills and duties: office workers (managers, professionals, technicians, and associate professionals), service and sales workers (clerical support, service, and sales workers), and manual workers (skilled agricultural, forestry, fishery workers, crafts and related trades, plant and machine operators and assemblers, and elementary occupations). Mean working hours were calculated, and the use of PPE was categorized as follows: Those who did not require it (no need), those who required it and always used it (need/wear), and those who required it but did not use it (need/no wear).

### Statistical analysis

Statistical analyses were performed using SAS statistical software (version 9.4; SAS Institute Inc., Cary, NC, USA). Chi-square tests were used to compare differences in baseline characteristics between the cases and controls. Odds ratios (OR) and 95% confidence intervals (95% CI) for headache/eyestrain according to occupational noise or vibration exposures were estimated using a logistic regression model that adjusted for age (continuous), sex, educational level, household income, work hours, shift work, and PPE status. Thereafter, the goodness of fit of the fully adjusted regression model was tested. Area under the receiver operating characteristic curve (AUROC) analysis was used to assess the effect of occupational exposure to noise and/or vibration on headache/eyestrain. AUROC analysis was also used to evaluate differences in the contribution of each of these occupational exposures to the risk of headache/eyestrain. The AUROC analyses were compared using the ROCCONTRAST option of logistic regression procedure of SAS. For all analyses, a two-tailed p value <0.05 was considered statistically significant.

## Results

Of the 25,751 participants, 4,903 had experienced headache or eyestrain during the preceding year (case group) while the other 20,848 had not (control group). Each variable was restructured into a categorical format as shown in the second column of Table 1. There were significant differences in age, education level, household income, occupational classification, shift work, occupational vibration exposure, and occupational noise exposure (p < 0.05) between the two groups. In contrast, no meaningful differences between the two groups were found in terms of sex, working time, and use of PPE. Age was divided into four subcategories; 20–29, 30–39, 40–49, and 50–65 years. In both groups, the older age subcategories had more participants than did the younger. The percentage difference between cases and controls within each subcategory was found to be highest in 20–29-year age group and lowest in 40–49-year age group.

**Table 1:** Baseline characteristics of study participants according to experience of headache/eyestrain.

		Headache / Eyestrain		
Characteristic		Yes, *n* (%)	No, *n* (%)	P-value
**Total**		4903 (19.0)	20848 (81.0)	
**Sex**	Men	4215 (18.9)	18030 (81.1)	0.3438
	Women	688 (19.6)	2818 (80.4)	
**Age (years)**	20–29	428 (14.8)	2470 (85.2)	<0.0001
	30–39	1051 (18.1)	4769 (81.9)	
	40–49	1696 (21.2)	6313 (78.8)	
	50–65	1728 (19.2)	7296 (80.8)	
**Education level**	Elementary school	156 (20.8)	595 (79.2)	<0.0001
	Middle school	356 (19.6)	1464 (80.4)	
	High school	1793 (15.9)	9472 (84.1)	
	University or higher	2598 (21.8)	9317 (78.2)	
**Household income**	1^st^ quartile	943 (15.9)	5007 (84.1)	<0.0001
	2^nd^ quartile	932 (18.7)	4064 (81.3)	
	3^rd^ quartile	1484 (19.7)	6049 (80.3)	
	4^th^ quartile	1544 (21.2)	5728 (78.8)	
**Occupational classification**	Office workers	2079 (23.4)	6821 (76.6)	<0.0001
	Service and sales workers	1627 (16.4)	8271 (83.6)	
	Manual workers	1197 (17.2)	5756 (82.8)	
**Working time (hours/week)**	<48	2492 (18.9)	10711 (81.1)	0.4877
	≥48	2411 (19.2)	10137 (80.8)	
**Shift work**	Yes	385 (21.5)	1409 (78.5)	0.0068
	No	4518 (18.9)	19439 (81.1)	
**PPE**	No need	3775 (18.7)	16383 (81.3)	0.0546
	Need/wear	1012 (20.4)	3960 (79.6)	
	Need/no wear	116 (18.7)	505 (81.3)	
**Vibration exposure** ^a^	None	2254 (18.6)	9900 (81.4)	0.0354
	Mild	1941 (19.3)	8113 (80.7)	
	Severe	708 (20.0)	2835 (80.0)	
**Noise exposure** ^a^	None	2140 (17.4)	10138 (82.6)	<0.0001
	Mild	2235 (20.4)	8704 (79.6)	
	Severe	528 (20.8)	2006 (79.2)	

^a^ Occupational exposure

PPE, personal protective equipment.

Education was divided into four subcategories according to the highest level of schooling; elementary school, middle school, high school, and university or higher. 1,793(15.9%) participants at high school level have experienced headache/eyestrain, and it is the lowest proportion compared with workers who experienced headache/eyestrain in other education level. Household income was divided by quartile. 1,544(21.2%) workers in the lowest household income group reported their headache/eyestrain experience, and it was the largest percentage compared with other household income group.

There was no statistically significant difference in the distribution of PPE subcategories between the case and control groups. Both occupational vibration and noise exposure variables were classified into three subcategories from “none” to “severe”. Both variables showed statistically significant differences in the distribution of these subcategories between the case and control groups.

Results of odds ratios and 95% CI for headache/eyestrain are presented in Table 2. Workers who had never been exposed to occupational vibration or noise were categorized into a group labeled “none”; this was used as the reference group in the logistic regression models. The OR between vibration exposure and headache/eyestrain was 1.08 (95% CI, 1.01–1.16) in the mild vibration exposure group, and 1.26 (95% CI, 1.14–1.40) in the severe vibration exposure group, with a statistically significant trend with increasing level of exposure (P for trend < 0.0001).

**Table 2:** Logistic regression results for headache/eyestrain according to occupational exposure.

Exposure	Headache/Eyestrain	
	OR (95% CI)	P for trend
**Vibration Exposure:**		<0.0001
**None**	1.00 (reference)	
**Mild**	1.08 (1.01–1.16)	
**Severe**	1.26 (1.14–1.40)	
**Noise Exposure:**		<0.0001
**None**	1.00 (reference)	
**Mild**	1.25 (1.17–1.34)	
**Severe**	1.41 (1.26–1.58)	

OR, odds ratio; CI, confidence interval; Model adjusted for age (continuous), sex, education, household income, occupational classification, work time, shift work, and personal protective equipment status.

Those in the mild noise exposure group had 1.25 (95% CI, 1.17–1.34) times the odds of having experienced headache/eyestrain compared with those in the no exposure group, while the OR was 1.41 (95% CI, 1.26–1.58) for the severe noise exposure group relative to the no exposure group. A statistically significant trend of increasing OR increments was observed (P for trend < 0.0001).

AUROC analysis was used to compare the predictive power of occupational vibration and noise exposure on headache/eyestrain (Table 3). Not exposed group was classified as the reference group. According to these analyses, the AUROC value of the reference group was 0.5697. The AUROC value obtained from the relationship between occupational vibration exposure and headache/eyestrain was 0.5744, while that from the relationship between occupational noise exposure and headache/eyestrain was 0.5806. Both AUROC values were higher than 0.5, indicating that the models of occupational vibration and noise exposure had more explanatory power than random ones. The predictive power increased when both noise exposure and vibration exposure were considered in combination: the AUROC value of that model was 0.5808, also presented in S1 Fig.

**Table 3:** Comparison of the predictive powers for headache/eyestrain according to occupational noise and vibration exposures.

Exposure	Headache / Eyestrain		
	AUROC	SD	P value
No exposure	0.5697	0.00142	(reference)
Vibration exposure	0.5744	0.00170	0.0030
Noise exposure	0.5806	0.00178	0.0005
Vibration plus noise exposure	0.5808	0.00177	0.0019

AUROC, area under the receiver operating characteristic curve; SD, standard deviation.

AUROC for headache/eye strain of none exposure model estimated using covariates as follow; age(continuous), sex, education, household income, occupational classification, work time, shift work, and personal protective equipment status, then added noise, vibration, and both exposure, respectively.

## Discussion and conclusion

Our results show that both occupational noise and vibration exposure are related to neurological symptoms such as headache/eyestrain. This study also suggests that occupational noise exposure is more closely related to these two symptoms than is occupational vibration exposure.

Previous studies have indicated a strong relationship between these two exposures and headache/eyestrain. Physiologically, noise signaling pathways consist of a direct pathway to the auditory cortex and an indirect pathway to the limbic system, autonomic nervous system, and neuroendocrine system that is closely associated with the development and control of stress. During the signaling process, noise can affect alertness, and motor performance and can induce a number of physiological, emotional, and behavioral reactions [19]. Also, noise may trigger headache by disturbing the neurovascular system or provoking abnormal muscular tension [20–23]. Spierings et al. showed that noise exposure is enough to precipitate headache, including migraine and tension type headaches [24,25]. They reported that patients exposed to noise had a higher prevalence of headache relative to the control group. Martin et al. experimentally demonstrated that noise might be a factor triggering headache[26]. The relationship between vibration exposure and headache has been thoroughly reviewed. Matoba et al. reviewed 300 cases of inpatients with vibration disease before and after treatment. In this study, patients suffered from central nervous system and higher center autonomic nervous system disorders, such as headache, palmar hyperhidrosis, forgetfulness, tinnitus, and impotence, when they were exposed to hand-arm vibrations (not to whole-body vibrations). Overall, 52.0% of patients in that study reported experiencing headache, which improved significantly after 3 months of treatment [27]. Thus, in addition to noise exposure, vibration exposure can unbalance the function of nervous system and cause physiological change [28]. Moreover, Harada et al. suggested that long-term exposure to vibration could result in hyperactivity of the sympathetic nervous system [29]. Tian et al. demonstrated that noise and vibration can hinder central nervous system function and worsen eyestrain, leading to impaired visual motor reaction time [30].

Apart from those studies, it is difficult to find a study that considers occupational noise and vibration exposure in combination. In the workplace, it is difficult to discriminate between these two exposures, because of their physical characteristics. Therefore, comprehensive approaches to assessing the combined effect of those two exposures are needed.

This study is among the first to demonstrate and compare the impact of occupational noise and vibration exposure, separately and in combination, on headache/eyestrain. We specifically assessed these exposures in combination; hence, the findings of our study can be applied to actual workplace environments more appropriately. The associations found in this study support an idea that comprehensive management of these two exposures is important for workers’ health. In addition, the gaps noted between the two groups in terms of the PPE subcategories “no-need” and “need/no-wear” suggest the need for urgent intervention.

In this study, the two occupational exposures to noise and vibration were investigated through a self-reported questionnaire survey. People are usually more sensitive to noise than vibration, so occupational vibration exposure might have been underestimated. Although this study suggests that occupational noise exposure has more potential to provoke headache/eyestrain, it is difficult to infer that managing vibration exposure is less important than managing noise exposure.

We used headache/eyestrain as the outcome variable in this study. Headache/eyestrain, a subjective symptom, is one of the most common complaints reported by patients [11]. Both headache and eyestrain are able to generate and aggravate annoyance, which can cause workplace accidents and injuries. Sadri et al. studied 219 bus drivers and suggested that the chance of accidents was significantly associated with migraine[31]. Wilkins et al., suggested that chronic conditions such as migraine were significantly associated with work-related injuries[32]. Moreover, headache not only degrades quality of life, but also leads to significant economic and financial loss. Migraines or chronic headaches are a leading cause of presenteeism which can decrease productivity[33].

The limitations of this study include the following: First, symptoms of headache and eyestrain were investigated by a self-reported questionnaire, so the study might not be free from self-reported bias and uncertainty. However, considering that previous studies found high agreement between self-reported symptoms and physician diagnosis, the reliability of this study can be preserved [34]. Second, subjective factors could have obscured the measurement of each exposure; underestimation or overestimation can occur depending on individual sensitivity [35]. Our questionnaire could not quantify the severity of noise exposure according to exact decibels or of vibration exposure according to measured amplitude, acceleration, frequency, or duration of vibration. Third, the KWCS was not customized for the purpose of this study, thus previous medical history, including past diseases or medication which could affect an individual’s sensitivity to occupational exposures, was not investigated. Some individuals who suffer from migraine or visual problems might be hypersensitive to noise or vibration [36,37] in the workplace; this could exaggerate the effect of occupational noise and vibration exposure on headache/eyestrain. However, we investigated a dose-response relationship between occupational exposure and headache/eyestrain, and the relationship was significant despite the absence of previous medical history data. Also, as this study was based on a cross-sectional study design, causality could not be established. Last, it would have been better if the survey had used a more specific questionnaire based on the International Classification of Headache Disorders (ICHD-3) criteria [38–40] or Migraine Disability Assessment Score (MIDAS) [41–43], to obtain detailed information about headache/eyestrain. To overcome those limitations, future longitudinal prospective studies using specific and clinically meaningful questionnaires and accurate measurement of exposures could be considered.

In conclusion, we found the relationship between occupational noise and vibration exposure and headache/eyestrain; noise exposure has greater influence. When the two exposures are considered in combination, the explanatory power for headache/eyestrain is increased. Using these findings, future studies can be designed to clarify the relationship between occupational noise and vibration exposure and headache/eyestrain with more scientific programmed surveys or specific quantification. Moreover, to improve workers’ health and the workplace environment, continuous efforts aimed at reducing and managing occupational noise and vibration exposures are crucial, and remain a work in progress.

## Supporting information

S1 Fig ROC curves for comparisons between occupational noise and vibration exposures. (TIF)

S1 File Dataset used in this study; from the fourth KWCS database. (SAS7BDAT)
