# Supplementary figures and images for: The relationship between occupational noise and vibration exposure and headache/eyestrain, based on the fourth Korean Working Condition Survey (KWCS)
Source: PLoS One. 2017 May 23;12(5):e0177846. doi: 10.1371/journal.pone.0177846 (PMC5441589; doi:10.1371/journal.pone.0177846)

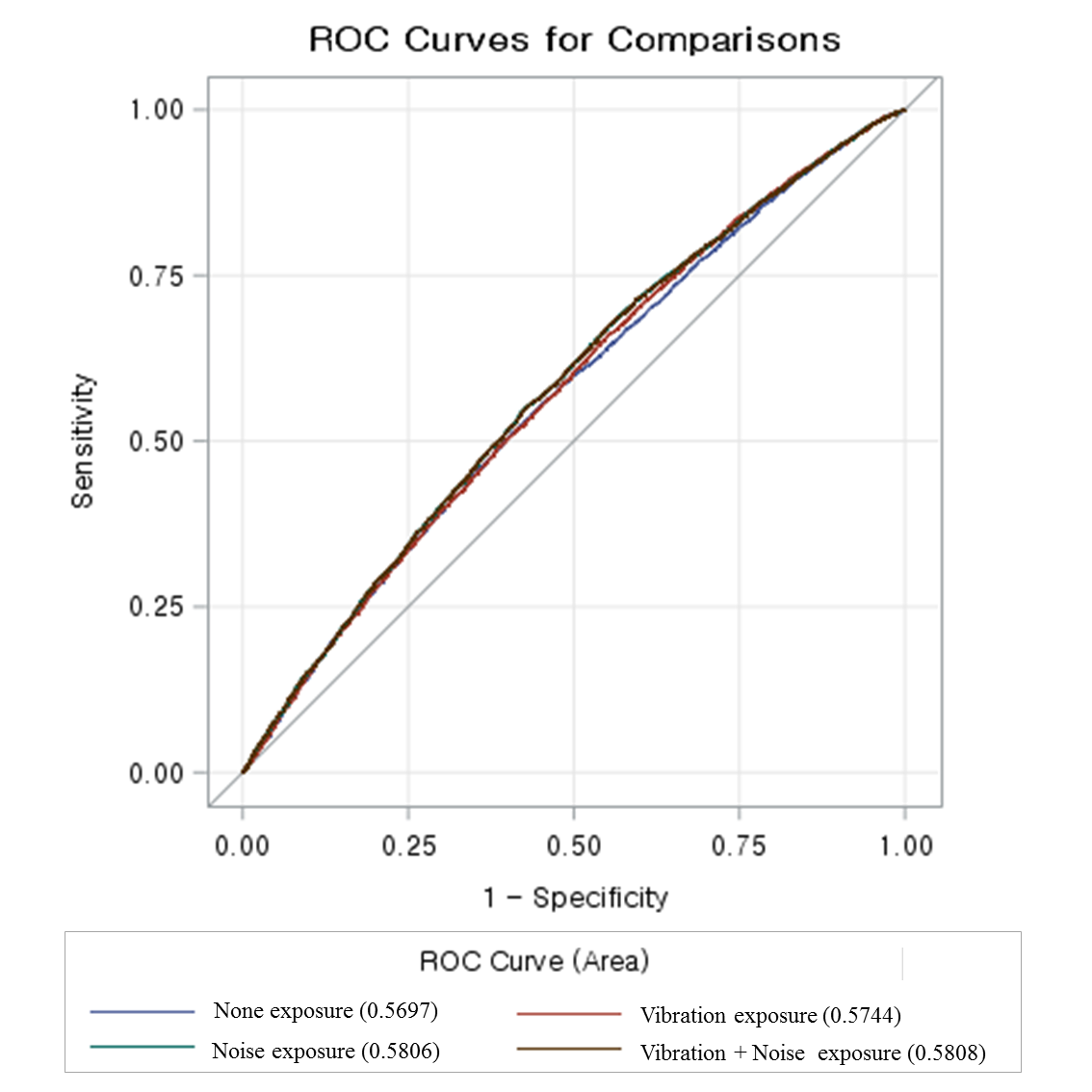

Supplement: S1 Fig — (TIF) [file pone.0177846.s001.tif]
